# Light-curing units in restorative dentistry: a clinically oriented narrative review of performance, selection, and emerging optical functions

**DOI:** 10.1038/s41405-026-00446-9

**Published:** 2026-05-20

**Authors:** Romain Ceinos, Margaux Dubois, Jean-Pierre Attal, François Vigouroux, Yohann Flottes, Timothy Fasham, Elisabeth Dursun, Bruno Pelissier, Maria-Antonietta D’Agostino, Philippe François

**Affiliations:** 1 Department of Conservative Dentistry and Endodontics, Côte d’Azur University, Nice, France; 2 INSERM UMR1333 Oral Health, Faculty of Dental Surgery, Université Paris Cité, Montrouge, France; 3 UPR7354 Oral Microbiology, Immunotherapy and Health, Côte d’Azur University, Nice, France; 4 Department of Dental Materials, Faculty of Dental Surgery, Université Paris Cité, Paris, France; 5 Private Practice, Cadaujac, France; 6 Department of Prosthetic Dentistry, University Paris-Cité, Paris, France; 7 Department of Paediatric Dentistry, University Paris-Cité, Paris, France; 8 Department of Conservative Dentistry and Endodontics, University of Montpellier, Montpellier, France; 9 Department of Rheumatology, Catholic University of Sacred Heart, Foundation Policlinico Universitario Agostino Gemelli IRCCS, Rome, Italy

**Keywords:** Bonded restorations, Composite resin

## Abstract

**Objective:**

To synthesize current evidence on dental light-curing units (LCUs), focusing on their radiometric performance, clinical selection criteria, and emerging optical functionalities.

**Methods:**

This narrative review was conducted in accordance with the SANRA recommendations. A structured search of PubMed/MEDLINE was performed to identify publications addressing the technological evolution of light-curing units, the parameters guiding device selection, clinical optimization and safety considerations, as well as the integration of fluorescence, transillumination and beam-shaping adjunctive features within contemporary systems.

**Results:**

LED LCUs have become the clinical standard, evolving from monowave to polywave devices to better match both Norrish type II and type I photoinitiators. Across technologies, the effectiveness of curing depends on the delivered radiant exposure, spectral compatibility, beam homogeneity and effective tip diameter rather than the manufacturer-reported irradiance. Clinical determinants (distance, angulation, maintenance, battery state and training) strongly modulate the delivered energy. Thermal and ocular hazards must be addressed through controlled protocols and protective measures. Multifunctional LCUs integrating fluorescence-aided identification and transillumination functions and interchangeable tips may support diagnosis and workflow optimization, but independent evidence remains limited.

**Conclusions:**

Curing should be approached as controlled energy delivery. Reliability depends on the radiant exposure, spectral compatibility and beam geometry rather than the nominal irradiance. Third-generation polywave LED LCUs appear to be the most versatile reference option for routine practice, particularly when the photoinitiator system of the material is unknown or includes Norrish type I initiators. The clinical value of integrated diagnostic and beam-shaping functions remains to be independently validated.

## Introduction

Light-polymerizable adhesive/resin composite systems constitute the most widely used material combination for direct restorative procedures [1–3]. Limiting the discussion to adhesive and resin composite materials would be reductive, even though they are the most widely used resin-based materials. Other formulations, such as resin-based luting agents and resin-modified glass-ionomer materials, are also light-activated [4, 5]. This highlights the importance of the appropriate use of light-curing units (LCUs) in restorative dentistry, particularly as some authors have suggested that the polymerization quality may be more critical to clinical success than the specific material or technique selected to construct the restoration [6].

However, despite its recognized scientific importance, light-curing is often considered a simple, time-consuming procedural step [7–9]. In reality, it represents a critical energy-delivery process required to activate the various photoinitiator systems incorporated into modern resin-based materials, thereby initiating efficient photoactivated free-radical polymerization and allowing the material to achieve its intended chemical, mechanical, biological, and esthetic properties [7–10].

With technological refinement, light-curing technologies have progressed from quartz–tungsten–halogen units to plasma arc systems, lasers, and successive LED generations. However, the performance of such technology does not depend solely on the light source [7–9]. Belonging to a given technological category does not guarantee an equivalent curing efficiency, as significant variations may exist between individual LCUs of the same type [7–9]. Optical parameters such as collimation, the light guide design, the effective tip diameter, the spectral distribution, and beam homogeneity are crucial, as devices with similar nominal radiant exitance values may distribute energy differently and lead to localized undercuring, especially in posterior areas and deep proximal boxes [11–13]. In addition, the effectiveness of light-curing is strongly dependent on operator-related factors, including proper positioning of the LCU tip, maintenance of the minimal distance and correct angulation, adequate training and optimized protocols to ensure consistent and sufficient radiant exposure at the restoration surface [14–16]. Moreover, the safety of both the patient and the practitioner must be considered, as an excessive thermal rise within the pulp‒dentin complex and repeated ocular exposure to high-irradiance blue‒violet light represent clinically relevant risks that require controlled energy delivery and appropriate protective measures [17–19].

More recently, LCUs have progressively evolved toward multifunctional optical devices that integrate or interface with diagnostic and adjunctive technologies rather than function solely as photopolymerization units [20]. Some contemporary systems incorporate modified light guides, specific emission modes, or interchangeable tips designed to adapt light delivery according to particular clinical objectives [20].

Together, these developments suggest that LCUs cannot be selected according to only the manufacturers’ reported irradiance values [7–9]. A meaningful evaluation requires consideration of their spectral compatibility, beam profile characteristics, clinical ergonomics, safety parameters, and the potential integration of adjunctive optical features [7–9]. In accordance with the SANRA recommendations for narrative reviews [21], this clinical-oriented narrative review aims to synthesize current evidence on light-curing units, focusing on their radiometric performance, clinical decision-making, and emerging optical functionalities relevant to restorative dentistry.

## Materials and methods

### Search strategy

This narrative review was prepared using the SANRA items as a practical quality checklist for narrative reviews. The literature search was conducted in PubMed/MEDLINE on February 9, 2026, and included publications from January 1, 2000, to the date of the search. This temporal restriction was introduced to ensure consistency with the scope of the present review, with a focus on the evolution of light-curing technologies, the parameters underlying device selection, and their integration with optical diagnostic tools and adjunctive features in restorative dentistry. It also allowed the exclusion of earlier studies that, although historically important, are less directly aligned with the objectives of this analysis. This choice was also informed by the large number of publications identified during initial, nonstructured exploratory searches performed while developing the project.

Keywords were developed through iterative exploratory searches and refined after reviewing the terminology used in key publications within each thematic area. Particular attention was given to variations in terminology across successive generations of light-curing technologies, key performance descriptors (including irradiance, radiant exposure, spectral emission, and beam profile characteristics), and the various optical diagnostic tools and adjunctive features integrated within these systems, such as fluorescence-based approaches, near-infrared transillumination, modified light guides, interchangeable curing tips, beam-shaping optics, filters, and dedicated accessories designed to optimize light delivery. Additional terms were identified on the basis of the authors’ clinical and research experience in adhesive and restorative dentistry.

Three separate PubMed search equations were therefore used to explore (1) the evolution of light-curing technologies, (2) parameters relevant to device selection and the optimization of their clinical use, and (3) the integration of optical diagnostic tools and adjunctive features within contemporary curing systems, in accordance with the structural framework of this narrative review. The search equations are presented in Table 1. In addition to database searching and citation tracking, some publications were included because they were considered essential to the conceptual framework of the review on the basis of the authors’ expertise and critical reading of the field. The final reference list comprised 181 publications. Of these, 128 (70.7%) were selected after full-text assessment of records retrieved through the three structured PubMed search equations, whereas 53 (29.3%) were added through backward and forward citation tracking, expert-informed selection, or contextual inclusion. These additional references were primarily incorporated in Sections “Light-Cured Resin Composites for Adhesive Luting” and “Integration of Adjunctive Optical Features”, which were not directly covered by dedicated search equations or due to the lack of standardized terminology and their more transversal nature beyond strictly defined photopolymerization-related concepts.

**Table 1:** Thematic PubMed search equations and number of retrieved records.

Search Focus	PubMed Search Equation	Number of Results
Equation 1 Evolution of light-curing technologies	(“Curing Lights, Dental”[MeSH Terms] OR “dental curing light” OR “light curing unit”) AND (evolution OR development OR progress OR advances OR trends OR generations OR comparison OR transition OR “state of the art”) AND (halogen OR “quartz tungsten halogen” OR QTH OR LED OR “light-emitting diode” OR “plasma arc” OR xenon OR laser OR ultraviolet OR UV) AND (“2000/01/01”[Date - Publication] “2026/02/09”[Date - Publication])	342
Equation 2 Selection criteria	(“Curing Lights, Dental”[MeSH Terms] OR “dental curing light” OR “light curing unit”) AND (criteria OR selection OR performance OR efficiency OR optimization OR evaluation) AND (irradiance OR intensity OR “radiant exposure” OR wavelength OR spectrum OR spectral OR “beam profile” OR collimation OR distance OR thermal OR temperature OR “pulp temperature” OR photoinitiator OR monowave OR polywave) AND (restorative OR “restorative dentistry” OR “composite resin” OR “resin cement”) AND (“2000/01/01”[Date - Publication] “2026/02/09”[Date - Publication])	443
Equation 3 Integration with optical diagnostic tools	(“Curing Lights, Dental”[MeSH Terms] OR “dental curing light” OR “curing light” OR “light curing unit”) AND ((fluorescence OR autofluorescence OR “fluorescence-aided” OR “fluorescence aided identification” OR FIT OR QLF OR “Quantitative Light-Induced Fluorescence”[MeSH Terms]) OR (transillumination OR “Transillumination”[MeSH Terms] OR “fiber optic transillumination” OR FOTI OR DIFOTI OR “near infrared transillumination” OR “near-infrared transillumination” OR NIR OR NILT OR DIAGNOcam) OR (“light guide” OR “light guides” OR “curing tip” OR “curing tips” OR “light tip” OR “light tips” OR “light pipe” OR “light pipes” OR “optical fiber” OR “fiber optic” OR “Optical Fibers”[MeSH Terms] OR lens OR filter* OR collimat* OR attachment* OR accessory* OR “tip diameter” OR tapered) OR (integrat* OR integrated OR multifunction* OR multimode OR “dual mode” OR combined OR convergence)) AND (restorative OR “restorative dentistry” OR “dental restoration” OR “Composite Resins”[MeSH Terms] OR “composite resin” OR “resin composite” OR “resin cement” OR luting OR adhesive) AND (“2000/01/01”[Date - Publication] “2026/02/09”[Date - Publication])	328

The selected publications were analyzed narratively, focusing on the progressive evolution of curing technologies, the parameters directly influencing the performance of light-curing units, the integration of optical diagnostic tools and adjunctive features, and the areas that remain controversial in the literature.

### Inclusion and exclusion criteria

Eligibility criteria were defined prior to screening to ensure consistency with the scope of the review and its predefined thematic structure (Sections “Evolution of Light-Curing Technologies”, “Selection Criteria and Clinical Use of Light-Curing Units” and “Integration of Adjunctive Optical Features”). Titles and abstracts retrieved on the basis of the three searches were screened for relevance in relation to these themes. Articles that were considered potentially relevant underwent a full-text assessment. The title and abstract screening and the full-text assessment were conducted by a single author in accordance with the predefined eligibility criteria. No formal duplicate screening, inter-rater cross-check, or inter-rater agreement calculation was performed. When the relevance of a publication was unclear, the decision was made according to the predefined thematic framework and its contribution to the clinical narrative.

#### Inclusion criteria

Studies were considered eligible if they met the following criteria:

- Addressed chairside dental light-curing technologies in the context of restorative, prosthetic or adhesive dentistry.
- Examined the technological evolution of light-curing units, their clinical performance or the safety determinants influencing light-curing choice or use, and/or the integration of optical features and diagnostic modalities associated with curing devices.
- Were published in English between January 1, 2000, and February 9, 2026.

#### Exclusion criteria

Articles were excluded if they did not meet the predefined inclusion criteria or were redundant with other articles that were already cited and had greater contributions. The overall identification and selection process using Zotero software (Corporation for Digital Scholarship, Falls Church, Virginia) is summarized in Table 2.

**Table 2:** Overview of the article identification, screening, and inclusion process.

Inclusion steps	Source	Action	Number of Articles
1	Equation 1	Articles obtained from Equation 1	342
2	Equation 1	Articles considered potentially relevant after title and abstract screening	199
3	Equation 2	Articles obtained from Equation 2	443
4	Equation 2	Articles considered potentially relevant after title and abstract screening AND after removing duplicates already retained according to Equation 1.	300
5	Equation 3	Articles obtained from Equation 3	328
6	Equation 3	Articles considered potentially relevant after title and abstract screening AND after removing duplicates already retained according to Equations 1 and 2.	311
7	All equations	Articles retrieved from Equations 1, 2, and 3 were selected for inclusion based on their relevance to the review’s objectives after an integral read, with consideration given to maintaining a coherent and focused reference list.	128
8	Additional studies	Additional articles identified through backward and forward citation tracking and expert-informed selection.	14
9	Additional studies	Additional articles used for contextualization, introduction, and specific sections, voluntarily excluded from the search equations or included despite not being identified through the predefined search strategy.	39
10	Total	Total articles included in the narrative review (Step 7 + Step 8 + Step 9).	181

### Data extraction

Data extraction was performed using a working document in Microsoft Word. Rather than using a separate spreadsheet, the selected articles were organized directly within the structure of the manuscript according to the predefined sections of the review (Sections “Evolution of Light-Curing Technologies”, “Selection Criteria and Clinical Use of Light-Curing Units” and “Integration of Adjunctive Optical Features”). This allowed the literature to be integrated progressively as the manuscript was developed.

For each article, bibliographic details and the main characteristics of the study are noted in the relevant section. When certain aspects were unclear, the full text was revisited to ensure accurate interpretation. Given the narrative nature of this review, no formal risk-of-bias tool was applied. Instead, the relevance and weight of each publication were considered in accordance with the study design, the quality of reporting, and its applicability to restorative dentistry.

## Results

### Evolution of light-curing technologies

#### Radiometric parameters and photoinitiator systems

To ensure terminological consistency throughout this review, key optical and curing parameters are defined in accordance with the 2021 FDI Policy Statement [22], Price et al. [7] and Neumann et al. [23]. The following definitions are adapted from these documents and include the relevant units of measurement where applicable:

- Radiant exitance (mW/cm²): Radiant power emitted from a surface per unit area.
- Irradiance (mW/cm²): Radiant power incident upon a surface per unit area. Irradiance is typically measured at specified distances from the light source. At 0 mm from the tip, irradiance corresponds to radiant exitance.
- Radiant exposure (J/cm²): The radiant energy delivered per unit area. This metric corresponds to the product of the irradiance (mW/cm²): and exposure time (s) divided by 1000.
- Emission spectrum (nm): The range of wavelengths emitted by a given light source.
- Spectral radiant power/spectral flux (mW/nm): Radiant power emitted, transmitted, reflected, or received per unit wavelength interval.
- Spectral irradiance (mW/cm²/nm): Irradiance expressed per unit wavelength interval, which describes the distribution of incident radiant power as a function of wavelength.
- Light beam homogeneity: The degree of homogeneity of both the radiant exitance and the spectral radiant power across the emitted light beam.
- Photoinitiator: A chemical species present in light-curable resin-based materials that, when exposed to light of a suitable wavelength, produces reactive radicals that can start the polymerization reaction.
- Photosensitizer: A molecule that absorbs light and, in the presence of a coinitiator such as a tertiary amine, participates in the formation of reactive species responsible for initiating polymerization.
- Molar extinction coefficient (L.mol⁻¹.cm⁻¹): The molar extinction coefficient (ε), also referred to as the molar absorptivity, is a constant that quantifies the intrinsic ability of a chemical species to absorb light at a specific wavelength. It reflects how strongly a molecule absorbs incident radiation at a given wavelength.

The primary role of an LCU is to induce the polymerization of light-curing resin-based materials [7, 9]. Dental adhesives, resin composites, resin-modified glass-ionomer cements and resin-based luting agents polymerize via photoactivated radical reactions to create a cross-linked polymer network from monomeric components [10]. This reaction is induced by a photoinitiator that initiates the polymerization process when it absorbs energy from a specific wavelength delivered by the LCU [10, 23].

The photoinitiators used in dentistry can be classified as belonging to either the Norrish type I or the Norrish type II family, depending on their curing mechanism [10]. The most well-known photoinitiator used in almost all dentistry resin-based materials is camphorquinone (CQ), which acts as a photosensitizer in a Norrish type II photoinitiator system. Norrish type II systems require interactions between a photosensitizer and a coinitiator (typically a tertiary amine) to generate free radicals that initiate polymerization [10]. This process consumes the C=C double bonds of dimethacrylates and leads to the formation of a crosslinked polymer network. CQ has a broad absorption spectrum in the visible range, extending from approximately 400 to 500 nm, with a maximum absorption peak at approximately 468 nm [10, 24]. At this wavelength, its molar extinction coefficient is relatively low, which means that an adequate number of photons must reach the material to efficiently activate the photopolymerization process [10, 23].

In contrast to Norrish type II photoinitiators, Norrish type I photoinitiators are directly cleaved upon light absorption, resulting in the direct generation of free radicals without the need for a coinitiator [10]. Compared with CQ, this unimolecular mechanism, which involves molecules with greater molar extinction coefficients, generally results in higher concentrations of generated free radicals and faster polymerization kinetics [10, 25]. The most widely used Norrish type I photoinitiators include trimethylbenzoyl-diphenylphosphine oxide (TPO), phenyl-propanedione (PPD), and germanium-based bis-acylphosphine oxide derivatives such as Ivocerin, whose molar extinction coefficient is greater than that of camphorquinone [10, 23]. Considering the toxicological concerns and recent regulatory scrutiny associated with TPO, this photoinitiator may progressively be replaced in future resin-based formulations by ethyl (2,4,6-trimethylbenzoyl) phenylphosphinate (TPO-L) [26]. From a regulatory perspective, this potential shift should be interpreted in the context of the EU harmonized classification of TPO as a CMR category 1B substance under the CLP framework and the increasing regulatory scrutiny of such substances. However, it should be noted that, unlike in some cosmetic applications, no specific restriction currently applies to its use in dental materials, although its presence in medical devices is subject to benefit–risk justification under the EU Medical Device Regulation (MDR). Compared with Norrish type II photoinitiators, Norrish type I photoinitiators absorb light at shorter wavelengths, with maximum absorption peaks at approximately 380 nm for TPO, approximately 410 nm for PPD, and 400–420 nm for Ivocerin [10, 24]. The introduction of Norrish type I photoinitiators was driven by the need to increase the reactivity, shorten the curing time, and increase the conversion rate, particularly in deeper or more opaque restorations [10, 25]. Their incorporation also facilitated the popularization of bleach and lighter shades, as they impart less yellow coloration to the resin composite compared with camphorquinone and reduce the risk of discoloration associated with the presence of amine coinitiators in Norrish type II systems [10, 25].

Therefore, the polymerization efficiency depends on the spectral compatibility between the LCU emission and the absorption characteristics of the photoinitiator system [7, 10, 23].

#### High-irradiance curing technologies: plasma arc and laser systems

To reduce the clinical time needed for light-curing of resin composites, high-irradiance “speed-curing” technologies were proposed to achieve reduced curing times. Additionally, quartz–tungsten–halogen (QTH) lamps were developed, which are described below [7–9]. These high-irradiance “speed-curing” LCUs are represented by plasma arc curing (PAC) units and lasers [7–9].

PAC units were introduced to the market with high radiant exitance generated by a xenon arc lamp that was filtered to deliver a broad blue spectrum (with limited violet emission in some cases) from approximately 400–500 nm [27]. Although this spectral range provides sufficient overlap to excite CQ-based Norrish type II photosensitizer/coinitiator systems, these LCUs were developed when CQ was the only initiator system in most direct resin-based dental formulations [1, 9, 28]. Compared with QTH or LED LCUs, these units were reported to induce a lower degree of conversion and reduce the depth of curing, even when the delivered radiant exposure was similar [29–31]. Moreover, some studies have suggested that PAC may increase marginal leakage despite claims of improved curing potential in clinical scenarios involving regions that are difficult to access with increased LCU tip distances [32, 33].

Lasers, such as argon and, more recently, diode LCUs, represent another category of high-irradiance curing units [34]. They can achieve, under some controlled conditions, polymerization outcomes similar to those of QTH and LED units [35–37]. Moreover, their high collimation, a property inherent to laser technology, is theoretically advantageous as the distance between the LCU and the restoration increases. However, although lasers can deliver very high radiant exitance at the tip, short-curing-time protocols may lead to insufficient radiant exposure at the restoration surface. This property, combined with a small and inhomogeneous beam profile, resulted in a reduced depth of curing across multiple resin composites containing Norrish type I photoinitiators and Norrish type II photosensitizer/coinitiator systems [38, 39].

In any case, for both PAC systems and lasers, in addition to their photopolymerization and clinical limitations, economic and practical factors restrict their widespread adoption [7, 9, 40]. PAC units are considerably more expensive than QTH and later-developed LED units are, while laser-based systems also involve safety constraints associated with the use of high-powered beams [27, 40].

#### Quartz–tungsten–halogen units and the emergence of LED technology

QTH units remained the most widely used LCUs in dental offices until the early 2000s, notably because they were more affordable and more practical than lasers or PAC units were [28, 41]. Their operating principle is relatively simple: light is generated by passing an electrical current through a tungsten filament enclosed in a quartz bulb filled with halogen gas [41]. The resulting emission arises from incandescence and is therefore inherently broad in terms of the spectral distribution. Although the emission encompasses the visible range, it also includes a substantial infrared component and, depending on the optical configuration, minor near-UV emissions [41, 42]. Because this emission is nonselective, significant internal optical conditioning is needed before the light can be used in clinical settings [41]. A reflector is used to concentrate and redirect the radiation toward the exit window, while bandpass and heat-absorbing filters are interposed to preferentially transmit the blue‒violet wavelengths effective for most dental photoinitiators and to attenuate the infrared radiation responsible for the thermal increase [41]. The spectrally conditioned output is then delivered through a fiber-optic guide to the restorative material [41]. This emission spectrum provides sufficient spectral overlap to initiate both the Norrish type I photoinitiators and Norrish type II photosensitizer/coinitiator systems used in contemporary resin-based material formulations [10, 23, 25, 42]. From an energetic perspective, however, QTH systems remain relatively inefficient. A considerable part of the electrical energy used is converted into heat rather than a useful optical output, which explains the need for active cooling systems. Despite their presence, these cooling mechanisms do not fully prevent the progressive thermal stress on the internal components, leading to bulb degradation, filter aging, and the need for regular maintenance and periodic replacement [9, 28].

In this context, gallium nitride-based blue LEDs were developed in the mid-1990s with a fundamentally different technological approach. Compared with QTH units, these LEDs had reduced heat generation and a narrower spectral bandwidth centered near the main absorption peak of CQ [7–9, 28]. Nevertheless, the first attempts to develop LED LCUs often resulted in devices that were less clinically efficient than QTH units, with limited radiant power, longer exposure times, and higher initial costs [9, 28, 43]. Nevertheless, these first-generation LED LCUs demonstrated that adequate polymerization could be achieved without the mechanical constraints and maintenance issues inherent to QTH technology [9, 28].

#### Generational evolution of LED light-curing units

Although the classification of LED LCUs into generations has not been fully standardized in the literature, the first-, second-, and third-generation terminology is used here as a didactic framework rather than as a formal taxonomy. It helps summarize the technological evolution that resulted in LED devices being established as the gold standard for light-curing resinous formulations in dentistry [28]. As reported in the previous section, first-generation LED devices produced limited radiant exitance with a narrow emission spectrum centered on CQ, which led some authors to refer to these units as “monowave” LED devices [7–9, 28, 41].

The transition to second-generation LED LCUs marked a major refinement in the field, and these new units progressively replaced the previously described systems in routine practice. Although these second-generation LED LCUs were still monowave devices, the multiemitter arrays used in the first generation were replaced with high-power blue diodes, resulting in a drastic increase in the radiant exitance at the LCU tip [7–9, 28, 41]. Under adequate radiant exposure, these second-generation LED LCUs were shown to achieve polymerization outcomes comparable to or better than those of QTH devices for CQ-based resin composites [43–49] while limiting clinical curing times, maintenance requirements and cost-related limitations associated with QTH units [9, 28, 50]. Importantly, with the parallel development of new resin composite formulations, including TPO, PPD, and Ivocerin, these second-generation monowave LED LCUs were, in a few studies but not all, unable to achieve meaningful degrees of conversion [44]. These observations are still strongly debated in many publications [51–54].

**Fig. 1:**
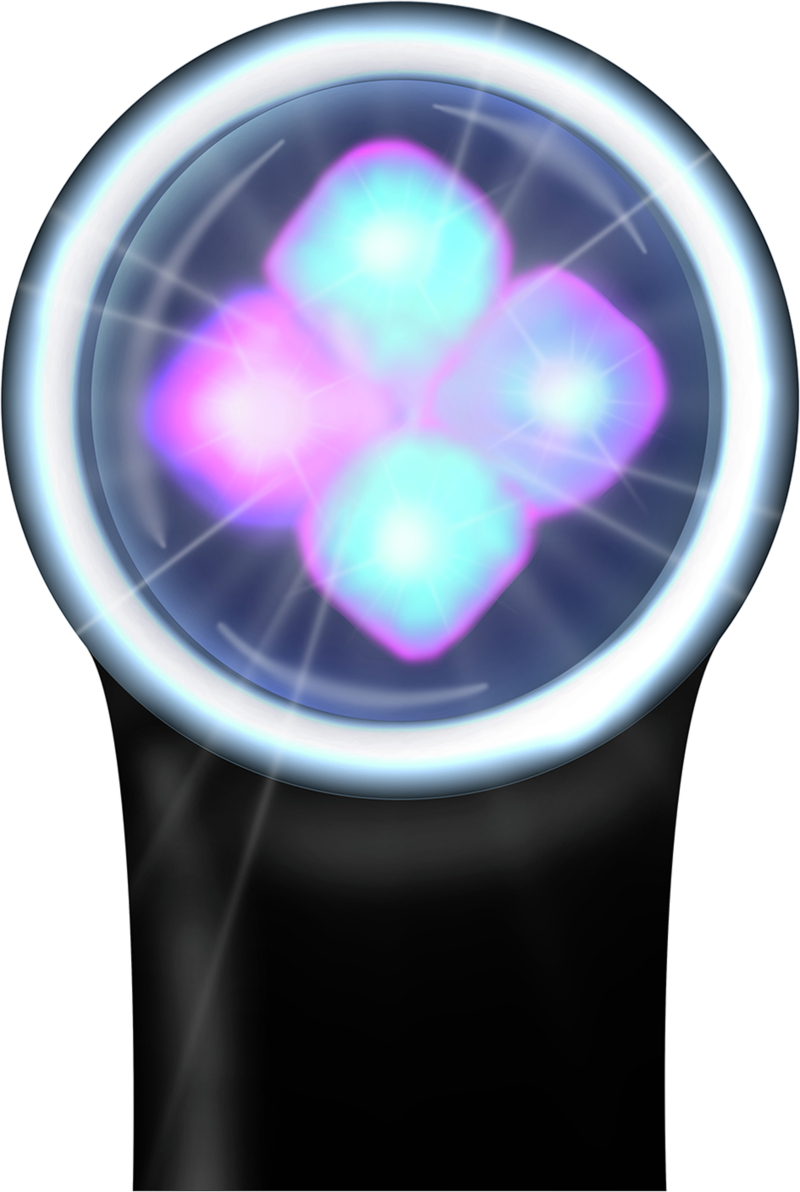
Schematic representation of a polywave LED LCU. This representation is inspired by the Valo LCU (Ultradent, South Jordan, UT, USA), illustrating four LEDs integrated within the same light head one emitting light at 405 nm (violet), one emitting light at 440–445 nm (intermediate blue), and two emitting light at 460–470 nm (blue).

This potential limitation in adequately activating the contemporary Norrish type I photoinitiators incorporated into many modern resin composite formulations served as a rationale for the development of third-generation “polywave” LED LCUs that emit both violet and blue light [11, 28, 55]. These third-generation LED units incorporate multiple narrow emission peaks in the violet (≈395–415 nm) and blue regions (≈450–470 nm) (Fig. 1) while maintaining or even increasing the high radiant exitance at the LCU tip provided by second-generation devices [52, 56, 57].

By extending the emission spectrum to the violet range, the spectral compatibility of these devices with Norrish type I photoinitiators is improved, and the effective activation of CQ-based systems, which remain present in most resinous formulations, often in synergistic combination with Norrish type I initiators, is maintained [52, 56, 57]. Compared with second-generation LED LCUs, different methods involving third-generation LED LCUs have achieved improved photopolymerization outcomes when resin composites containing both Norrish type I and type II photoinitiator systems are cured [56–64]. However, as briefly mentioned earlier, this superiority must be interpreted carefully [51–54]. When CQ-dominant resin composites are cured and when exposure protocols result in similar radiant exposure, second- and third-generation LED LCUs have demonstrated comparable degrees of conversion and mechanical properties in some studies [56, 65]. In such cases, the principal advantage of the higher-output third-generation units lies in their ability to deliver equivalent radiant exposure with shorter exposure times rather than their fundamentally different polymerization mechanisms [52, 56, 57]. Finally, shorter wavelengths, such as violet light, tend to penetrate less deeply into resin composites than blue wavelengths do because of Rayleigh scattering. This phenomenon should also be considered when a polywave LCU is used [12, 66–68].

#### Critical appraisal of contemporary LED generation

**Fig. 2:**
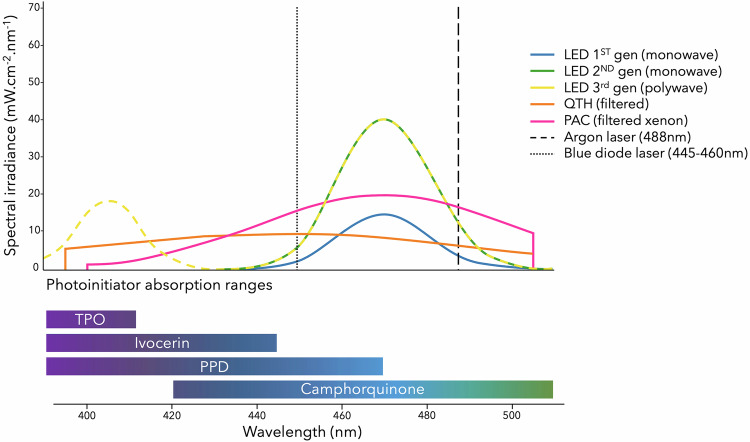
Schematic spectral emission profiles of dental light-curing technologies, expressed as spectral irradiance (mW·cm^−2·^nm^−1^) measured at 0 mm from the light-guide tip. Narrow-band LEDs have higher peak spectral irradiance than broad-band QTH and PAC sources, whereas polywave LEDs emit both violet and blue light. Laser sources are highly monochromatic and the peaks are clipped for readability. The spectral profiles are schematically illustrated for comparative purposes. The curve amplitudes are scaled to typical peak spectral irradiance values derived from representative radiant exitance and emission bandwidths and do not represent device-specific measurements. Inspired by the work of Rueggeberg et al. [8].

On the basis of the bibliographic analysis conducted in this review, the evolution of LCUs can be understood as a progressive transition from broad-spectrum but energetically inefficient sources such as QTH units and high-irradiance yet expensive and technically constraining attempts to shorten the curing time (PAC systems and lasers) toward more ergonomic and less expensive LED devices capable of delivering controlled radiant exposure and emission spectra that are more aligned with the Norrish type I and Norrish type II photoinitiator systems found in resin-based materials, particularly in the case of third-generation polywave LED units (Fig. 2 and Table 3) [7–10, 22, 23, 28, 42].

**Table 3:** Evolution of LCUs over time and their principal characteristics.

Light-curing unit type	Approximate introduction	Emission spectrum (nm)	Typical exposure time (s)	Compatible photoinitiators
QTH (Quartz-Tungsten- Halogen)	1980s	≈ 400–500 (after optical filtering)	20–40	Camphorquinone + spectral overlap with some Norrish type I initiators
PAC (Plasma Arc Curing)	1990s	≈ 400–500 (blue, limited violet in some devices)	5–10	Camphorquinone + spectral overlap with some Norrish type I initiators
Laser (argon)	1990s	≈ 488 nm (narrow band, quasi monochromatic)	5-20	Camphorquinone
Laser (diode)	Early 2000s	≈ 445–460 nm (narrow band, quasi monochromatic)	1-10	Camphorquinone
LED First-generation (monowave, low power)	Early 2000s	≈450–490 (peak ≈470)	20–30	Camphorquinone
LED Second-generation (monowave, high power)	Late 2000s	≈450–490 (peak ≈470)	10–20	Camphorquinone
LED Third-generation (polywave, high power)	2010s	≈380–515 (violet + blue peaks)	3–10	Camphorquinone + Norrish type I initiators (TPO, PPD, Ivocerin)

Note The LED “generation” terminology is used here as a didactic framework and is widely adopted in the literature to describe the technological evolution of light-curing units. However, it is not fully standardized, and individual devices may not fit neatly into a single category, as emission spectrum, radiant power, beam profile, and optical design vary across manufacturers.

Although the real clinical utility of third-generation polywave LED units compared with that of second-generation monowave LEDs with increased irradiance remains debated by some authors [51–54], recent systematic reviews and meta-analyses of in vitro studies tend to support a modest advantage of third-generation polywave units for the direct measurement of the degree of conversion and indirect assessments through hardness measurements, especially in resin-based materials incorporating Norrish type I and type II photoinitiators [69–71]. In this context, polywave LED units represent a marginal but clinically useful refinement of second-generation monowave systems rather than a true technological advance. Notably, the reported benefit seems more pronounced for resin composites than for adhesive systems [53, 70]. This may be explained by formulation differences between the two materials: most contemporary adhesives, including many recent universal systems, rely predominantly on camphorquinone as their principal photoinitiator, whereas many modern resin composites incorporate mixed Norrish type I/type II photoinitiator systems [1–3].

Importantly, neither high-irradiance settings nor polywave emission profiles eliminate the need for adequate radiant exposure at the restoration surface [11, 72, 73]. Beam profiles remain heterogeneous, violet wavelengths attenuate more rapidly with increasing depth, and clinical variables, including operator positioning, tip angulation, distance, and exposure time, as discussed in the following section, often appear more decisive than the mere choice between a second-generation monowave LED and a third-generation polywave unit [7, 9, 16].

Considering these limitations, until long-term clinical outcomes demonstrate robust equivalence among the diverse modern resin-based materials, third-generation polywave LED LCUs can reasonably be considered the most versatile and conservative reference option for routine light curing [69–71].

### Selection criteria and clinical use of light-curing units

#### Spectral compatibility and radiant exposure

When an LCU is chosen, spectral compatibility and energy delivery must be considered in addition to the choice of the resin-based material. The resin-based material can be efficiently polymerized only if the LCU emits light within the absorption bands of the photoinitiator system and delivers sufficient radiant exposure to the surface [7, 22, 23]. Thus, when selecting an LCU, discussing irradiance alone is less relevant than considering the radiant exposure delivered to a restoration: for a given LCU, the radiant exposure can be increased by extending the curing time, thereby increasing the total energy delivered to the restoration [74]. As discussed previously, the risk of spectral mismatch is not merely theoretical for resin-based materials and is more important for resin composites than for adhesives [1–3]. On the basis of these properties, as discussed in Section “Evolution of Light-Curing Technologies”, LCUs with a broad emission spectrum that can activate both Norrish type I and type II photoinitiator systems should be used. Therefore, in accordance with the currently available technologies, third-generation polywave LED units are recommended [69–71].

Importantly, even with efficient spectral compatibility, the clinical success of the final restoration ultimately depends on the radiant exposure received by the resin-based material. The irradiance values provided by manufacturers are often measured directly at the tip of the LCU, without clearly specifying the emitting surface area or the spectral composition of the measured beam. Thus, the given irradiance represents a global value [42, 75]. Notably, many high-irradiance “boost” modes, when considering the recommended exposure times, may deliver a radiant exposure to the restoration that is significantly lower than that achieved with standard modes, resulting in a less adequately polymerized resin-based material [11, 72, 73]. Consequently, given that this does not induce excessive heating of dental tissues (as discussed in Section “Thermal and Biological Safety Considerations”) [76], the curing time should be increased in the absence of precise knowledge regarding the specific LCU/resin-based material combination used, regardless of the curing mode selected on the device [56, 76–78].

Finally, the recent introduction of ultrabroad-spectrum polywave LED LCUs incorporating additional LEDs emitting in the red and near-infrared ranges should be discussed [79–81]. Theoretically, these additional wavelengths do not improve the polymerization of the Norrish type I or type II photoinitiators currently present in resin composites, although longer wavelengths to the red region are known to penetrate deeper into resin-based materials and dental tissues [10, 82]. Unsurprisingly, the results reported with these devices appear comparable or even slightly inferior to those obtained with conventional polywave LED units [72, 79–81]. However, the development of such devices could represent a potential future research direction if manufacturers were to commercialize photoinitiators absorbing in the red spectrum, although their integration, particularly from a colorimetric standpoint, would likely remain complex [72, 83].

#### Optical design and beam characteristics

**Fig. 3:**
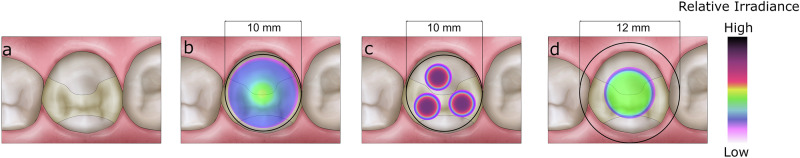
Schematic illustration of how beam homogeneity and effective tip diameter may influence light delivery to a mesio-occluso-distal restoration on a premolar. **a** Reference clinical situation. For illustrative purposes, the LCUs shown in (b–d) are assumed to have the same nominal irradiance. The black outline represents the external diameter of the LCU head (schematic, not to scale), whereas the color map represents the beam profile, i.e., the relative irradiance distribution. **b** LCU with an external head diameter close to the effective tip diameter and a moderately inhomogeneous beam profile, resulting in incomplete coverage of the mesial and distal boxes of the mesio-occluso-distal restoration. **c** LCU with poor collimation and marked beam inhomogeneity, generating localized hotspots and under-irradiated areas that are difficult to visualize or control clinically. **d** Theoretical LCU with a perfectly homogeneous beam profile, shown only to isolate the effect of a small effective tip diameter relative to the head diameter despite uniform irradiance within the beam footprint, substantial parts of the mesial and distal boxes remain outside the illuminated area. Overall, this schematic emphasizes that an idealized LCU would combine high beam homogeneity with the largest possible effective tip diameter, ideally close to the external diameter of the LCU head, so that the beam footprint can be anticipated more accurately clinically. Inspired by the work of Price et al. [7].

In addition to the spectral emission and radiant exposure, the optical architecture of the LCU is essential for determining how homogeneously radiant energy is delivered at the surface of and deeper in resin-based materials [11, 84, 85]. Two LCUs delivering a similar radiant exitance at the tip may induce markedly different polymerization patterns (Fig. 3). In practice, this is not a marginal issue. The effective emitting area, beam collimation, divergence, spatial distribution of the radiant exitance across the tip and spectral homogeneity across the emitted field are therefore critical parameters, and these values are sometimes more than the nominal output values would suggest [11, 84, 86].

An analysis of different beam profiles reveals that contemporary second-generation monowave LCUs and third-generation polywave LCUs may exhibit inhomogeneous radiant exitance distributions across the emitting tip [11, 84, 86]. This appears to be partly linked to the asymmetric positioning of the violet and blue LED chips within the light head (Fig. 1) and represents a distinction among devices [61, 67, 85]. Importantly, such spatial variations cannot be captured by conventional dental radiometers [87, 88]. As a result, they are frequently underestimated in both research settings and routine clinical practice.

**Fig. 4:**
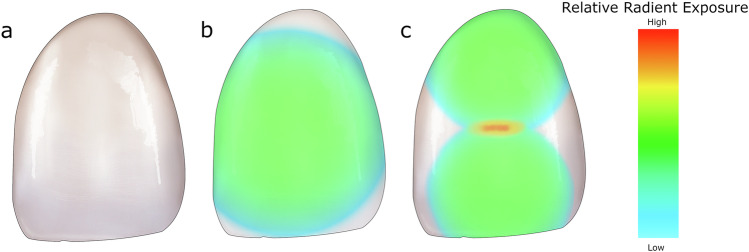
Schematic illustration of the influence of effective light-curing unit LCU tip diameter on the distribution of radiant exposure during light curing of a large direct resin composite veneer on a maxillary central incisor (mesiodistal width ≈9 mm; inciso-cervical height ≈11 mm). **a** Reference clinical situation. **b** Single curing position the effective beam footprint does not fully cover the restoration surface for most, if not all, currently available LCUs, including those with relatively large tip diameters, resulting in reduced radiant exposure at the peripheral areas. **c** Two overlapping curing positions using an LCU with a smaller effective tip diameter overall coverage is improved, but the overlap zone receives greater cumulative radiant exposure, while some peripheral areas may still receive insufficient energy for adequate polymerization. This schematic highlights that, for large anterior restorations such as a maxillary central incisor veneer, restoration dimensions may exceed the effective tip diameter of contemporary LCUs, so complete and homogeneous coverage generally cannot be achieved with a single curing position. Multiple curing positions are therefore required. The same principle also applies to large indirect restorations during adhesive luting, although the total delivered radiant exposure should be controlled to limit intrapulpal temperature rise in vital teeth. Color maps are schematic and do not represent device-specific measurements. Inspired by the work of Peres et al. [90].

The tip diameter and effective emitting area are also highly important [16, 39, 89]. The use of large-diameter tips (not to be confused with the external size of the LCU head, with the effective emitting surface being consistently smaller, albeit to variable degrees) may increase surface coverage in both the anterior (Fig. 4) and posterior areas [16, 39, 89]. However, LCUs with large-diameter tips require greater total radiant power to maintain an equivalent radiant exitance. In this context, the distinction between radiant power and radiant exitance is clinically relevant since an insufficient surface coverage can lead to heterogeneous polymerization in restorations exceeding the beam diameter [86, 90]. In daily practice, this becomes particularly relevant for wide posterior restorations [7, 16] or when luting indirect restorations with light-cure resin cements [91–93].

Notably, higher-end LED LCUs often incorporate larger effective tip diameters (and greater radiant power), which is generally advantageous from a clinical perspective in terms of surface coverage and light-curing safety [94]. However, such devices may not be available in all clinical settings or health systems. In these situations, comparable clinical outcomes may be achieved by adapting the curing protocol, for example by increasing exposure time and using multiple overlapping curing positions.

The optical design also impacts the divergence of the beam. Some LED units generate relatively collimated beams, maintaining a more stable irradiance at clinically relevant distances, whereas other units exhibit more rapid dispersion, enlarging the beam footprint but reducing the irradiance density as the distance from the restoration increases [84, 95]. This limitation is less pronounced in laser LCUs, which produce highly collimated and narrow beams [38]. Their limited coverage area, however, may render them less practical in routine clinical situations, at least for larger restorations [38].

Taken together, these findings indicate that a reliable optical design is critical to the polymerization of resin-based materials. Such a design determines how radiant energy is spatially delivered before any clinical variable, such as distance or angulation, is introduced. The homogeneity of the beam, effective emitting area, intrabeam spectral distribution and divergence behavior are intrinsic device characteristics that directly influence curing reliability [16, 42]. For this reason, LCUs should be selected according to not only nominal irradiance values but also their independent and scientifically validated optical performance. The selection of devices with optimized beam homogeneity and sufficiently large effective tip diameters appears to be, in this context, both logical and clinically justified [16, 42]. Similarly, in the case of clinical uncertainty regarding the adequacy of the photopolymerization of a resin-based material, particularly when the effective tip diameter does not fully cover the restorative surface, it appears reasonable to perform a second curing cycle from a different irradiation position [89, 90].

#### Clinical determinants of effective light delivery

**Fig. 5:**
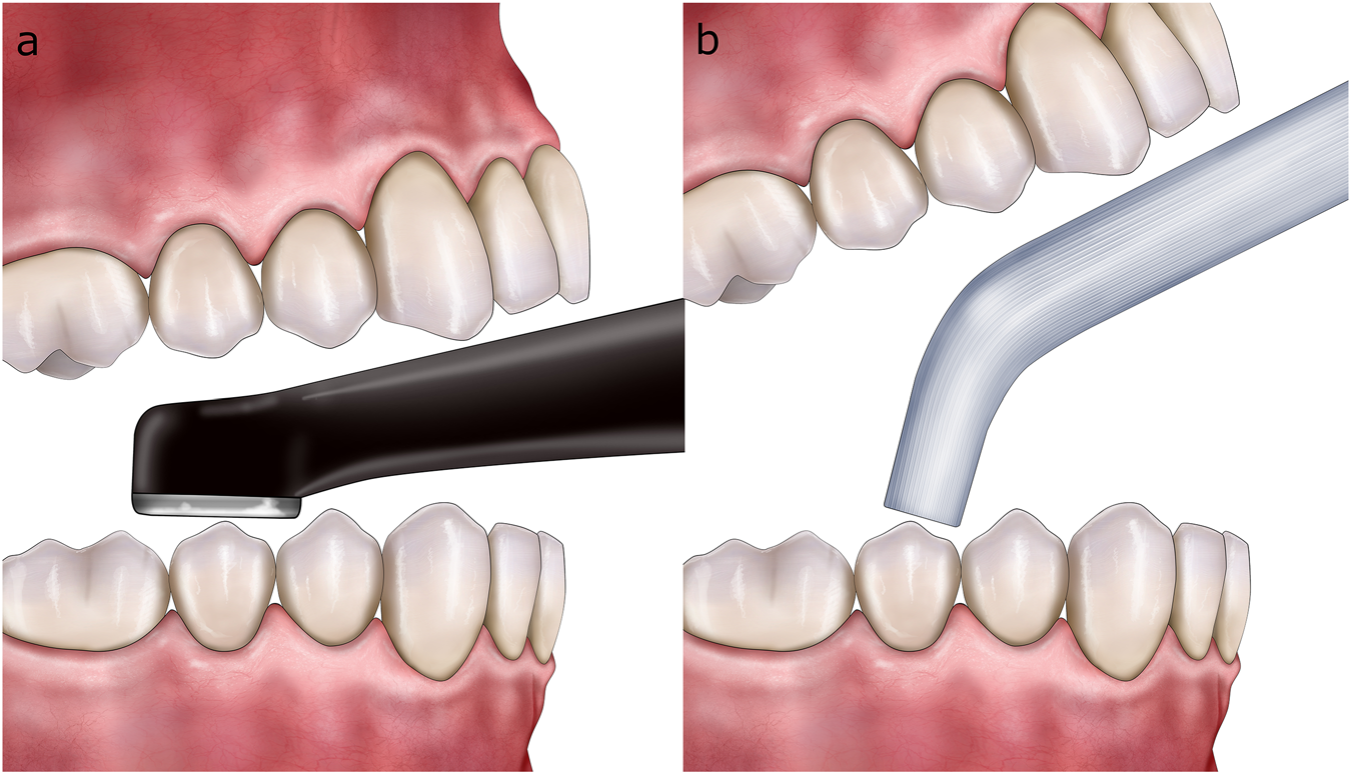
Schematic illustration highlighting the importance of light-curing unit (LCU) head design and access for effective photopolymerization of posterior restorations. Efficient light curing requires positioning the LCU tip as close as possible to the resin-based material and as perpendicular as possible to the restoration surface. **a** In a situation with sufficient physiological mouth opening, adequate access allows optimal positioning of the LCU head relative to the restoration. **b** In more constrained clinical situations, effective photopolymerization would require a mouth opening exceeding the patient’s physiological limits (a limitation that is even more pronounced in posterior molar regions). Under normal clinical conditions, the LCU tip cannot be ideally positioned, leading to suboptimal light delivery at the restoration surface. Inspired by the work of Shortall et al. [9].

Even when the chosen LCU exhibits favorable characteristics, the amount of radiant exposure effectively delivered to the resin-based material is strongly influenced by clinical parameters (Fig. 5). The distance between the LCU tip and the restoration should be kept as close as possible to 0 mm [13]. As the delivered irradiance decreases with increasing distance between the LCU tip and the restoration, even clinically realistic distances of 2–4 mm can significantly affect the polymerization of the resin-based material [13]. This effect becomes particularly relevant with deep proximal boxes and posterior cavities, as the distance between the LCU tip and the gingival floor frequently exceeds the manufacturer’s recommendations [86, 96].

The angulation of the LCU tip relative to the resin-based surface also alters the radiant exposure received by the material. In scenarios involving limited access and suboptimal tip orientations, the delivered radiant exposure may be considerably reduced, highlighting the importance of using an LCU that allows perpendicular positioning of the light beam, even with posterior molars [86, 96]. Indeed, in posterior regions, and particularly for Class V restorations compared with occlusal Class I preparations [96], physical interference owing to adjacent teeth or soft tissues may compromise the ideal perpendicular positioning of the light tip. When combined with an increased distance, these factors may substantially reduce the radiant exposure in critical regions, particularly the gingival margins of Class II restorations [97].

In addition, the restorative material itself can influence internal light propagation owing to its microstructural composition and optical properties, including its translucency and wavelength-dependent transmittance [68].

#### Thermal and biological safety considerations

Radiant exposure must therefore be delivered with caution, as part of the delivered energy (and more marginally, the exothermic material-dependent polymerization reaction [17, 98–100]) can be converted into heat, resulting in temperature increases in dental and periodontal tissues [18, 80, 98]. Among all these tissues, the pulp‒dentin complex remains the most studied, with an intrapulpal temperature-rise threshold of approximately 5.5 °C that should not be exceeded to preserve cellular viability, as originally described by Zach and Cohen in 1965 [98, 101]. However, this long-established threshold has been questioned by numerous researchers, who have challenged its validity as an absolute value [18, 102]. In any case, the impact of LCU-induced heating is real and undeniable. Nevertheless, this heating is strongly modulated by several tooth parameters, particularly the remaining dentin thickness [98, 102] and, perhaps even more importantly, pulpal microvascularization, which contributes to dissipating the temperature increase [18, 103, 104]. This temperature increase is also modulated by two major operator parameters: the radiant exposure delivered to the tooth structure and the specific LCU used [18, 101, 102]. Thus, the most risky situation during photopolymerization is the application of a dental adhesive if the dentin thickness adjacent to the pulp is reduced [100, 105]. When pulpal microvascularization is simulated, to better simulate physiological conditions, the successive steps of resin-based material application should not exceed the 5.5 °C threshold, provided that the radiant exposure delivered to the dental tissue does not exceed the equivalent of two or three standard polymerization cycles when a third-generation polywave LED LCU is used [73, 106, 107]. These temperature increases are particularly important to consider in the case of deep cavities or cervical preparations, as the residual dentin thickness is reduced and access-related constraints often necessitate prolonged or repeated irradiation [107]. Nevertheless, the maximum temperature increase occurs during the application of the adhesive, and the temperature decreases as each successive layer of the resin-based material is applied. These layers act as insulating barriers and increase the overall thickness [17, 108]. In addition, the use of a simple air syringe appears to be an easy and effective approach to limit the increase in the pulp temperature. It can be applied without increasing the risk of contamination of the resin-based material being used [109, 110].

Thermal effects are not limited to the pulp. Soft-tissue burns may also occur, even when a rubber dam is in place [111]. In some situations, the dam may paradoxically become unfavorable because of light absorption phenomena that locally increase heat [111]. Ocular safety must also be considered [19, 112]. Blue and violet light in polywave LCUs can contribute to retinal hazard and should not be overlooked [19, 112]. Protective barriers should be changed for each patient to prevent cross-contamination and avoid the reduction in the irradiance delivered by the LCU because of degradation over time [113]. Finally, because the release of monomers into the patient’s oral cavity should be minimized for health reasons, a glycerin gel should be used as an air barrier before polishing to help prevent the formation of an oxygen-inhibited layer. This increases the degree of conversion at the surface over the first few microns and contributes to the safety of light-curing protocols, improved patient safety [114] and the final quality of the restoration [115].

#### Training, monitoring, and protocol optimization

The efficiency of light-curing also depends on practitioner training, quality control and monitoring of the LCU, and, for a given radiant exposure, the selection of appropriate curing modes according to the clinical situation.

Many studies have shown a lack of delivered information to practitioners and students during light-curing [14, 116, 117], and patient simulators have shown advantages in teaching practitioners how to use LCUs efficiently in specific clinical situations [14, 118]. This leads to increased delivered radiant exposure [14, 118]. These training programs, which show benefits over time [15], suggest that efficiency is also determined on the basis of practical training and the modification of various habits [118].

Quality control and monitoring are also essential, as damage can drastically decrease the irradiance delivered by an LCU tip [119]. Several studies have shown that a significant number of practitioners use LCUs with resin debris on light guides, chipped tips, or reused barrier sleeves [113, 119]. All of these factors have been shown to significantly reduce LCU output [119, 120]. For cordless LCUs, the battery level is another concern. Depending on the unit and its internal design, the irradiance may decrease as the battery charge decreases [121–124]. For a considerable number of devices, a lower battery level is indeed associated with reduced light output [121–124]. Routine inspection, cleaning, and replacement of damaged light guides are simple measures to preserve curing effectiveness [119]. In daily practice, this mainly involves careful visual checks of the unit and the regular use of the same radiometer. Although the absolute irradiance values provided by these devices may not be entirely reliable, as discussed above, repeated measurements allow comparisons over time and help verify that the LCU is functioning properly [87, 125].

Additionally, the light-curing protocol can be adapted to reduce the polymerization shrinkage stress induced by the photopolymerization reaction of resin-based materials. Even if this topic was more extensively studied in the past, the publications identified in this narrative review suggest that delaying the sol-gel point may help limit stress development during polymerization [45, 126, 127]. In this context, avoiding very high-irradiance modes during the initial curing phase may be important since shrinkage stress is strongly influenced by early reaction kinetics [45, 126, 127]. Similarly, when the LCU provides pulse or modulated modes, these approaches may contribute to reducing stress development, provided that the delivered radiant exposure remains adequate [128–131]. Nevertheless, some contemporary resin composite formulations have been developed specifically in accordance with the ultrashort light-curing protocols used with third-generation polywave LED LCUs [132, 133]. In this context, chain transfer-based approaches incorporating resin chemistry have been proposed to increase the reproducibility of high-irradiance curing approaches [132, 133]. This concept relies on specific resin composite formulations being used with specific LCUs, where the material and the curing mode are designed as a consistent system [132, 133]. Most studies evaluating this specific type of dedicated system suggest that compared with other protocols, high-irradiance, ultrashort protocols are more consistent, provided that the manufacturer’s curing conditions are used [132, 133].

Finally, the PubMed search conducted for this narrative review highlighted more marginal or original parameters that can influence the clinical use of LCUs. For example, a right-handed operator may receive higher radiant exposure when working on the right side of the mouth, whereas the opposite tendency has been reported for left-handed operators [134]. In addition, although dual-cure resin cements are widely used in prosthodontics, they still appear to benefit from an additional light-curing step whenever clinically feasible [135, 136]. Finally, when more saturated and highly pigmented shades are used, the light-curing time should be increased, consistent with the manufacturers’ instructions, as such pigments can reduce light transmission and internal light diffusion within the material [137].

#### Light-cured resin composites for adhesive luting

Light-cured resin composites have long been used as luting agents, particularly for partially bonded restorations. For years, this indication remained largely confined to flowable resin composites, with only sporadic proposals advocating the use of preheated viscous restorative resin composites to improve rheology [138–140]. However, these approaches, especially those based on preheated viscous resin composites, have recently re-emerged through two structured clinical concepts: the “no-finishing concept” [91] and the “multiluting concept” [92]. In the no-finishing concept, excess resin composite is shaped and removed while the material is still in the plastic phase, with the aim of minimizing or avoiding bur finishing at the margins and increasing the working time [91–93]. The multiluting concept extends this approach to quadrant rehabilitation: several indirect restorations are inserted under rubber dams and stabilized sequentially with short, localized light exposures before the final cure [91–93].

The safety of using preheated viscous resin composite with single-use compules or minimally used syringes is well documented [141, 142]. However, the issue of complete seating with these preheated high-viscosity formulations, especially for less experienced practitioners, has long been debated [143–145]. Nevertheless, the primary clinical concern is not related to insertion accuracy. The main issue involves the ability to achieve adequate light polymerization of the luting resin composite beneath the indirect restoration. This issue becomes particularly critical when restorations vary in thickness [121] and are fabricated from materials with heterogeneous optical properties and different levels of translucency [146–148]. Once a strictly light-cured restorative resin composite replaces a dual-cured resin cement as the luting agent, polymerization becomes entirely dependent on the radiant exposure actually delivered at the tooth resin composite interface.

In this context, the incident irradiance at the restoration surface is not a clinically relevant parameter [146–148]. What truly governs the degree of conversion is the transmitted radiant exposure, which depends on the spectral emission profile of the LCU, the attenuation through the restorative material, the geometry of the irradiation beam, and the exposure time. The diameter of the emitting tip of the LCU must also be considered, particularly for wide restorations. A tip smaller than the restorative surface may result in a radiant exitance distribution with substantial spatial heterogeneity, leading to uneven radiant exposure across the luting interface. When these preheated viscous resin composites are properly light-cured, they may even reinforce the mechanical strength of the bonded prosthetic material [138]. At this clinically sensitive interface involving the restoration thickness, material optics, and light delivery, the LCU selection and optimization of the polymerization protocols are particularly important. These concepts represent a clinically demanding scenario in which LCU selection and energy-delivery parameters become determinant rather than accessory.

**Fig. 6:**
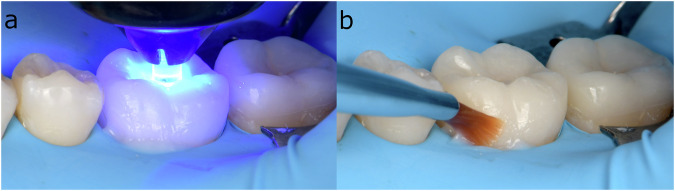
Main clinical steps of restoration assembly following the “no-finishing” concept. After the restoration containing preheated viscous resin composite is seated, a short-localized tack cure is performed with a collimating tip (PointCure, Ultradent) to stabilize the restoration while keeping the marginal excess partially uncured (**a**). Peripheral excess material is then removed with hand instruments and a brush lubricated with modeling resin (**b**) before prolonged light-curing is performed from multiple directions to ensure sufficient radiant exposure at the tooth–restoration interface.

In the “no-finishing concept” [91] and the “multiluting concept” [92], the protocol first relies on a brief, localized “tack cure” using a collimating tip on the LCU (Fig. 6a) to prevent polymerization at the margins and allow proper removal of excess material with hand instruments and a brush lubricated with a modeling resin [149] (Fig. 6b), followed by prolonged light-curing at multiple points of the restoration.

On the basis of the previously described selection criteria and clinical considerations for LCUs, several factors associated with the safety and optimization of such light-curing protocols can be discussed when luting indirect restorations with preheated viscous resin composites. Although the restorative resin composite is preheated prior to insertion, this step has only a limited effect on the increase in the intrapulpal temperature [150]. The temperature increase is governed mainly by the radiant exposure delivered during polymerization rather than by the initial temperature of the resin composite [150, 151]. Moreover, as the restoration thickness increases, its insulating effect becomes more pronounced, reducing heat transmission toward the pulp. Thicker restorations may therefore partially mitigate the increase in the pulpal temperature during extended irradiation [151].

In terms of the deep photopolymerization of the strictly light-cured viscous resin composites used as luting agents, the available in vitro data suggest that adequate polymerization can be achieved even beneath relatively thick restorations, including those composed of zirconia-based materials [146–148, 152–156]. However, the extent remains highly heterogeneous depending on the restoration thickness, material type and translucency, photoinitiator system, spectral emission profile, radiant exitance at the tip, tip diameter, and spatial distribution of the LCU [146–148, 152–157]. Experimental configurations and measurement methods further contribute to this variability. A consistent trend nevertheless emerges: with prolonged irradiation aimed at increasing the transmitted radiant exposure at the resin composite–tooth interface, satisfactory degrees of conversion may reasonably be obtained beneath restorations with thicknesses between at least 2 and 3 mm for most types of materials and translucencies [146–148, 156, 157]. Beyond this range, the predictability of the degree of conversion becomes increasingly material-dependent and sensitive to the characteristics of the LCU, including the tip diameter and beam homogeneity. These findings should be interpreted in light of the limited long-term clinical data currently available [158].

In any case, these protocols still lack standardization in terms of the light-curing strategy [91, 92]. There is no consensus regarding the minimal transmitted radiant exposure required at the interface, the optimal radiant exitance and tip geometry, or the ideal combination of the material, thickness, and irradiation strategy. In clinical settings, careful selection of the LCU is essential, and the spectral output, spatial homogeneity of the radiant exitance, tip diameter relative to the restoration size, and total radiant exposure delivered should be considered. Strategies to control pulpal heating, including a moderate irradiance during stabilization and active cooling during prolonged curing periods, should be integrated into the protocol [17, 108–110].

The main clinical implications of LCU selection and use are summarized in Table 4, with emphasis on spectral compatibility, radiant exposure, beam characteristics, clinical access, material-related attenuation, maintenance, safety, and operator-dependent factors.

**Table 4:** Practical clinical recommendations for light-curing unit selection and use.

Clinical determinant	Recommended clinical approach	When uncertainty or suboptimal conditions are present
Overall LCU selection	Do not rely on manufacturer-reported irradiance alone. Also consider emission spectrum, radiant exposure at the restoration surface, beam homogeneity, effective tip diameter, collimation, independent validation and ergonomics.	When only nominal irradiance is known, avoid assuming that shorter exposures are equivalent. Use conservative standard protocols until the material-LCU combination is validated.
Spectral compatibility and photoinitiator system	Identify the photoinitiator system when possible. CQ-dominant materials mainly require blue light, whereas materials containing Norrish type I initiators require violet/near-violet emission.	Use a polywave LED LCU when the photoinitiator system is unknown or when Norrish type I initiators may be present. A monowave LED LCU may be adequate for CQ-dominant materials if radiant exposure and beam coverage are sufficient. Increasing exposure time may partially compensate for suboptimal spectral overlap by increasing delivered radiant exposure, but it is unlikely to fully compensate for the absence of appropriate wavelength emission required to activate specific photoinitiators.
Radiant exposure and exposure time	Interpret curing as energy delivery per unit area rather than as a timed step. Radiant exposure depends on irradiance at the target surface and exposure time.	If compatibility, access or light transmission is uncertain, increase exposure time or repeat exposure within thermal safety limits rather than relying only on high-irradiance claims.
Curing mode	Use the standard curing mode as the default. Use boost, ultrashort, pulse or soft-start modes only when the material, LCU and protocol are indicated or independently supported.	For unfamiliar material-LCU combinations, avoid off-label ultrashort curing. A longer standard exposure is usually the more conservative option.
Beam homogeneity and effective tip diameter	Prefer LCUs with documented homogeneous radiant exitance and spectral distribution. Compare the effective emitting area, not the external head diameter, with the restoration dimensions.	If beam quality is unknown or the restoration exceeds the beam footprint, use multiple overlapping curing positions and include peripheral margins, proximal boxes and cervical areas.
Distance, angulation and clinical access	Keep the LCU tip as close as possible and as perpendicular as possible to the resin-based material, especially in posterior areas.	When increased distance, oblique angulation or limited access cannot be avoided, extend exposure time and/or cure from additional directions while considering heat generation.
Deep proximal boxes and posterior margins	Consider gingival floors of class II restorations and posterior margins as high-risk zones because distance, angulation and matrix obstruction reduce delivered radiant exposure.	Use careful tip positioning and additional occlusal, buccal or lingual exposures when feasible. Use light-transmitting adjuncts only as access aids, not as proof of increased delivered energy.
Material-related attenuation	Account for increment thickness, opacity, translucency, shade saturation, filler content and restoration geometry, all of which may reduce light transmission.	Follow material-specific increment and curing recommendations. Increase exposure time for opaque, saturated or highly pigmented shades and avoid extrapolating protocols across materials or shades.
Indirect restorations and light-cured luting	For strictly light-cured resin composites or resin cements used under indirect restorations, focus on transmitted radiant exposure at the tooth-restoration interface rather than irradiance at the external surface.	Use prolonged multipoint curing and active cooling during repeated exposures. If restoration thickness, opacity or photoinitiator compatibility makes light transmission uncertain, a dual-cure luting material may be safer.
Maintenance, barriers, monitoring and battery state	Inspect the LCU tip and light guide for debris, cracks or resin contamination. Use infection-control barriers correctly and consider battery state in cordless units.	Clean or replace damaged components, avoid degraded or repeatedly reused barriers, keep cordless units adequately charged, and monitor output over time with the same radiometer as a quality-control tool.
Thermal, soft-tissue and ocular safety	Balance longer or repeated exposures with pulpal, periodontal and soft-tissue heating. Use protective eyewear or shields suitable for blue-violet light and avoid direct viewing of the beam.	In deep cavities, cervical preparations or prolonged luting protocols, avoid unnecessary exposure, consider air cooling or cooling intervals, and maintain protective measures for both patient and operator.
Operator training and protocol standardization	Treat light-curing as a controlled clinical procedure. Operator positioning, stabilization of the LCU, angulation and distance should be trained and periodically reassessed.	Use standardized, procedure-specific curing protocols. In high-risk situations (e.g., indirect luting or deep posterior restorations), document the curing mode, exposure time, and irradiation strategy to ensure consistent and adequate energy delivery.

These practice-oriented recommendations synthesize the evidence and clinical considerations discussed in Section “Selection Criteria and Clinical Use of Light-Curing Unit”. They are intended to complement, not replace, manufacturer instructions and material-specific curing protocols.

*CQ* camphorquinone, *LCU* light-curing unit.

### Integration of adjunctive optical features

#### Conceptual basis for multifunctional integration

**Fig. 7:**
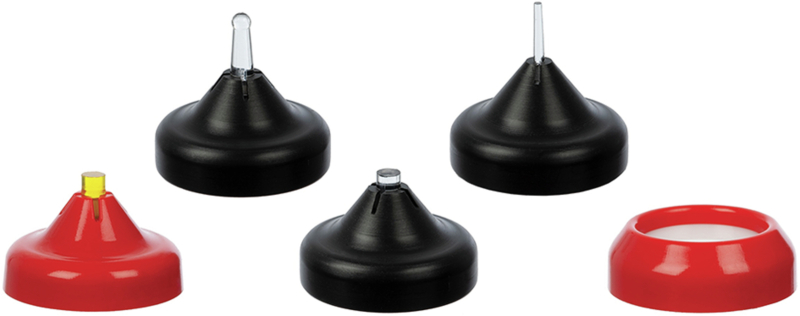
Examples of interchangeable optical accessories magnetically attached (also called lenses) to a LCU (VALO X, Ultradent). Depending on the accessory used, the same LCU can assist in transillumination procedures, selective localized curing, interproximal light delivery, or modification of the beam geometry. Used with permission from Ultradent.

Restorative dentistry has progressively evolved toward more minimally invasive strategies, reinterventions on existing restorations, and repair-oriented approaches. As a result, improved diagnosis methods and better control of the “invisible” aspects of clinical procedures, such as the residual material, marginal defects, cracks, and enamel defects, are needed [159]. Thus, with the evolution of LED LCUs, especially third-generation polywave LED LCUs, multiple spectral outputs have been integrated within the same handheld unit (for example, by adding LEDs emitting white light into the LCU head), either through built-in multimode emission strategies or through the use of interchangeable optical lenses and filters (Fig. 7) [7, 16]. This new type of LCUs is not merely a marketing trend: similar physical and optical principles are involved in designing a reliable LCU and an optical diagnostic device [160–163]. In other words, once an LCU is designed to deliver controlled light in the violet and blue ranges with a defined beam profile, it becomes technically feasible to repurpose or adapt this platform to deliver light in other spectral bands (near-UV excitation and visible-light transillumination) and to modify the illumination geometry for inspection rather than curing [160–164]. As a result, the field has progressively shifted from solely LCUs toward multifunctional optical platforms, in which photopolymerization remains the core function among several adjunctive features [160]. This integration also requires a clear distinction between diagnostic and curing functions. A device capable of switching between optical modes does not guarantee equivalent performance across all modes. On the clinical side, however, this integration may facilitate the wider adoption of diagnostic or operative techniques that are sometimes considered marginal but remain clinically relevant while maintaining financial accessibility and ergonomic integration within daily practice.

This narrative review also aimed to clarify the integration of these various adjunctive features into LCUs, as, to our knowledge, such a focused analysis has not yet been performed. Although appropriately centered, the specific PubMed search equation for this section, the snowballing approach based on backward and forward citation tracking of the identified articles, and manual research did not yield a substantial number of publications specifically addressing these LED LCU devices. This highlights a significant lack of independent studies on this topic, as well as a probable major gap in knowledge and potential regarding these affordable features, despite their conceptual and practical relevance. In this context, the present narrative review aims to bridge this gap by synthesizing the available information and presenting a structured understanding of these features. Nevertheless, because of the limited body of evidence, this section cannot reach the same level of evidentiary strength as the previous parts of the review. Therefore, a deliberately didactic and educational perspective is adopted to clarify the principles, possibilities, and practical implications of the adjunctive features offered by LCUs while preserving the clinical relevance of and interest in their integration. For clarity, manufacturer-specific color names are not used here as scientific descriptors. Here, the manufacturer-specific term “black light” is referred to as near-UV illumination. “White light” is described according to its clinical use, i.e., visible-light transillumination for inspection and filtered visible-light illumination for shade selection. Near-infrared transillumination is considered separately as a distinct diagnostic modality.

#### Fluorescence-based identification tools

Fluorescence is based on the ability of tissues or materials to emit secondary light at a longer wavelength after excitation at a specific shorter wavelength [164, 165]. In restorative dentistry, the diagnostic function of fluorescence involves two complementary phenomena. The first is the autofluorescence of hard dental tissues, which can change according to the mineral content and structure. The second is the fluorescence of restorative materials, which can be either intrinsic (related to the fillers and pigments) or intentionally enhanced to increase contrast under specific illumination conditions [164–167].

**Fig. 8:**
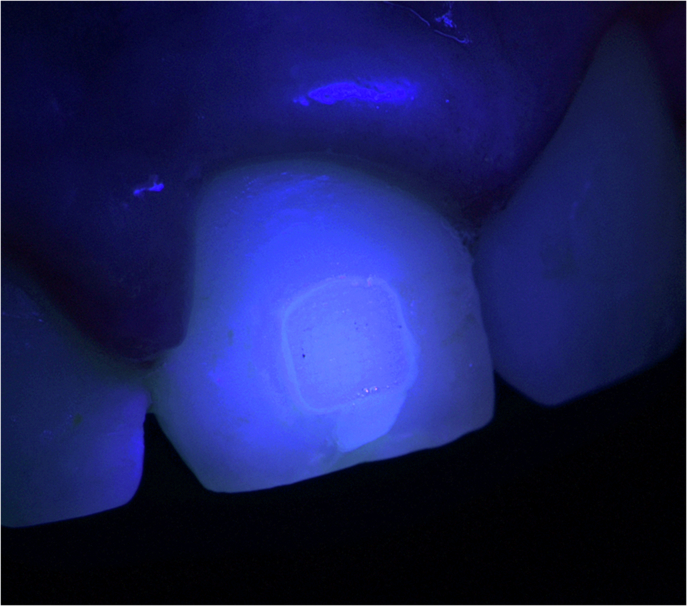
Fluorescence-aided identification of resin composite under near-UV illumination in an orthodontic context. Detection of residual orthodontic adhesive after bracket removal by a fluorescence-aided identification approach under near-UV illumination using a specific optical lens (Diffuser, Ultradent) magnetically attached to its associated LCU.

**Fig. 9:**
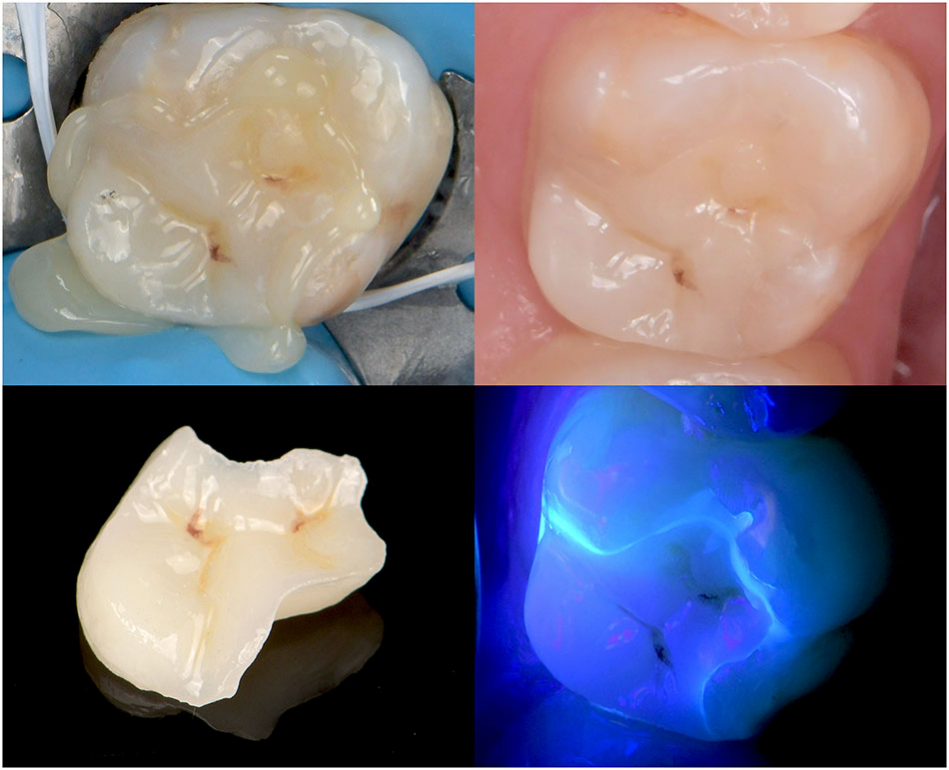
Fluorescence-aided identification of resin composite under near-UV illumination in a restorative context. Detection of excess resin cement associated with a posterior indirect partial restoration by a fluorescence-aided identification approach under near-UV illumination with the same optical lens and LCU than Fig. 8.

Several fluorescence-based strategies have been described in the literature. Quantitative approaches (such as quantitative light-induced fluorescence) aim to provide semiobjective information, mainly in the context of enamel demineralization and lesion monitoring. These techniques are not used with LCUs [165]. However, fluorescence-aided identification techniques that are fundamentally pragmatic have been proposed with some LED LCUs [166, 168–170]. This optical parameter is used to investigate tooth structure defects and the contrast between a fluorescent restorative material and the surrounding tooth structure to highlight defects that remain poorly visible under conventional operatory lighting conditions [168–171] (Figs. 8 and 9).

From a clinical perspective, this latter approach is particularly relevant for the replacement of tooth-colored restorations, the removal of orthodontic adhesive remnants, and the removal of excess resin cement after adhesive luting [169, 171–173]. In these situations, fluorescence-based visualization may help preserve sound enamel and reduce the risk of iatrogenic damage by limiting over-instrumentation and also lead to a gain of clinical time [174]. However, the fluorescence contrast is highly dependent on the spectral characteristics of the excitation source, the ambient lighting conditions, the viewing angle, and the fluorescence intensity of the restorative material itself [168].

#### Transillumination adjuncts and limited NIR integration

**Fig. 10:**
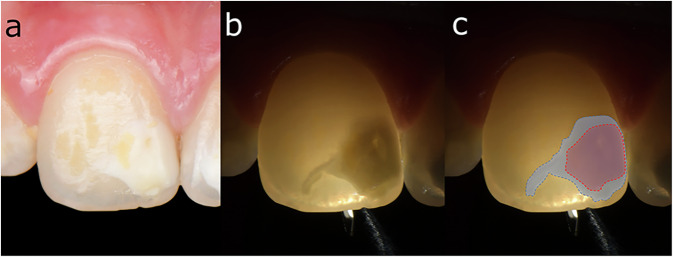
Assessment of the apparent depth of enamel hypomineralization in molar-incisor hypomineralization (MIH) using a specific optical lens (TransLume, Ultradent) with visible-light transillumination. **a** shows the initial clinical situation, **b** shows the visible-light transillumination view, and (**c**) shows a schematic map derived from the transillumination findings, with light blue indicating more superficial hypomineralization compared with red.

Transillumination is based on the propagation of light through dental tissues and restorations, with altered zones detected on the basis of changes in light scattering and absorption [167]. Unlike fluorescence, which relies on emission properties, transillumination relies on how light is attenuated and redirected within the tooth–restoration complex [167]. As a result, transillumination is particularly useful for detecting enamel cracks and proximal lesions, as well as for mapping structural defects that cannot be clinically detected under conventional lighting conditions [175–177] (Fig. 10).

In the context of contemporary light-curing units (LCUs), the adjunctive diagnostic applications identified in this review are primarily based on the transmission of visible light through dedicated lenses, modified light guides, or accessory optical elements. Such approaches may assist in the visualization of enamel cracks, the assessment of interproximal regions, and the monitoring of the lesion depth during minimally invasive procedures such as resin infiltration [20, 175–178]. Several studies have reported the usefulness of transmitted light for highlighting changes in enamel translucency and structural discontinuities, thereby supporting clinical decision-making in certain situations. However, the available literature mostly addresses transillumination techniques in general rather than functions specifically integrated into LCUs [20, 175–178].

Near-infrared-based transillumination represents a distinct diagnostic approach that has shown promise for the detection of early carious lesions and structural changes within dental tissues. Nevertheless, the studies retrieved in the present review primarily involved dedicated diagnostic systems rather than conventional LCUs. Consequently, although some emerging devices may incorporate broader spectral emission profiles [79–81], the integration of true near-infrared diagnostic capabilities into LCUs remains limited and insufficiently documented in the literature.

#### Adjunctive optical accessories for the curing of resin-based materials

The integration of specific accessories for the curing of resin-based materials is the final aspect considered in terms of the multifunctionality of LED LCUs. These adjunctive features include interchangeable lenses and light guides designed to shape, concentrate, diffuse, or redirect the emitted beam.

The first category consists of focused or collimating lenses or tips that can reduce the illuminated area to a narrow diameter and increase control when selective polymerization is desired [91, 92] (Fig. 6). Clinically, this is particularly relevant for stabilization steps in adhesive workflows, where a brief localized exposure is used to “lock” a restoration in position without fully polymerizing excess marginal material, thereby allowing careful clean-up prior to definitive curing, as in the no-finishing or multiluting concepts described above [91, 92].

**Fig. 11:**
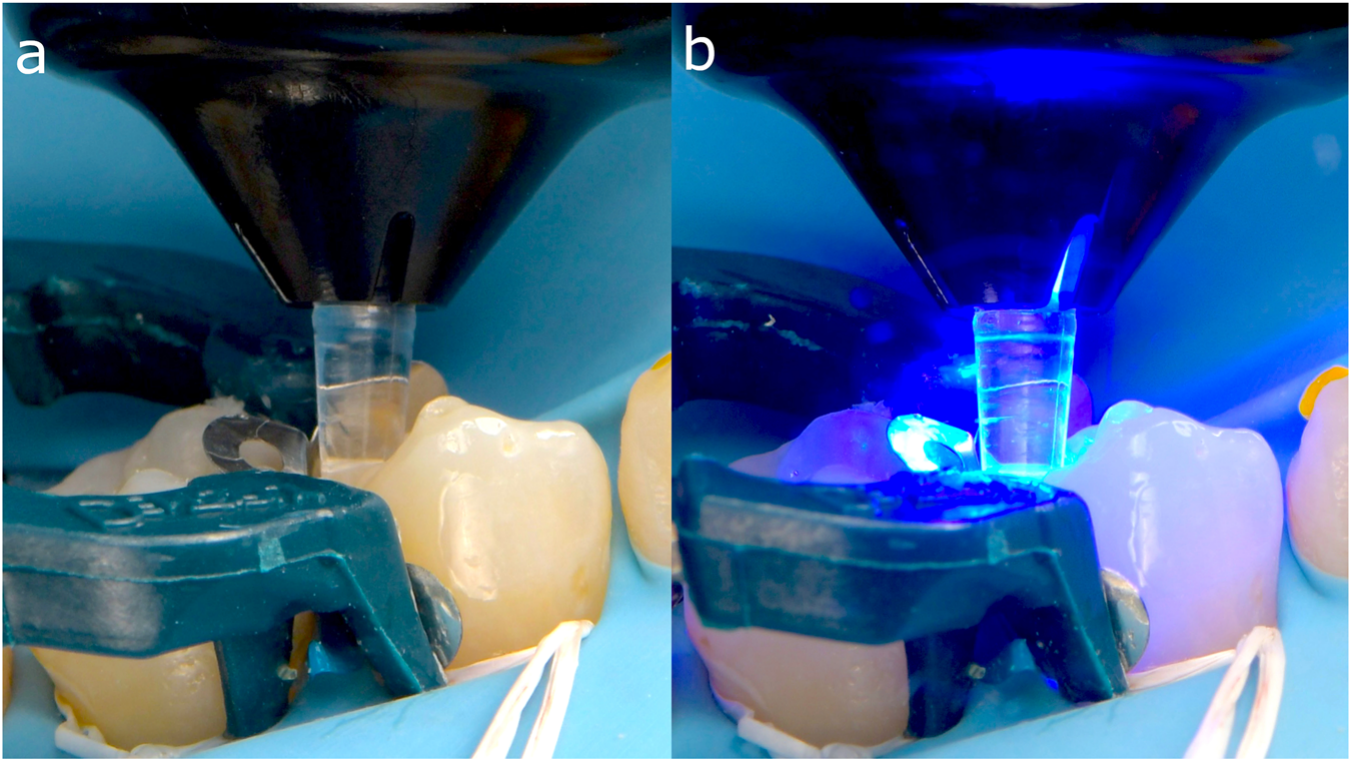
Use of an interproximal optical accessory lens (Proxicure Ball, Ultradent) to shape uncured resin composite against the matrix prior to light-curing and assist in proximal contact formation. **a** Light curing is performed with the accessory inside the class II cavity (**b**), which is then removed.

A second category is transproximal or interproximal curing assistance. Deep proximal boxes and contact areas are recurring sites of undercuring because of their distance from the LCU, their angulation, and the obstruction of light by the metal matrix [97]. Transparent contact-forming instruments and light-transmitting wedges have been proposed to shape proximal contacts while allowing curing through the instrument [179, 180]. Similarly, specialized proximal lenses or tips have been proposed as adjunctions of LED LCUs to deliver light laterally or to diffuse light in confined interproximal spaces [179] (Fig. 11). These approaches link mechanical and optical control: they aim to stabilize matrices, shape contact points of direct restorations, and increase radiant exposure in local regions. Although this concept is interesting, in reality, any intermediate medium introduces reflection and attenuation. Therefore, the benefits should be viewed in terms of increased access and geometry improvements rather than a guarantee of increased delivered radiant exposure. In contrast, dedicated light lenses and tips that aim to broaden or homogenize the emitted beam are also available. However, these devices inevitably lead to significant modifications in the radiant exitance distribution and the overall energy delivered at the target surface [161].

**Fig. 12:**
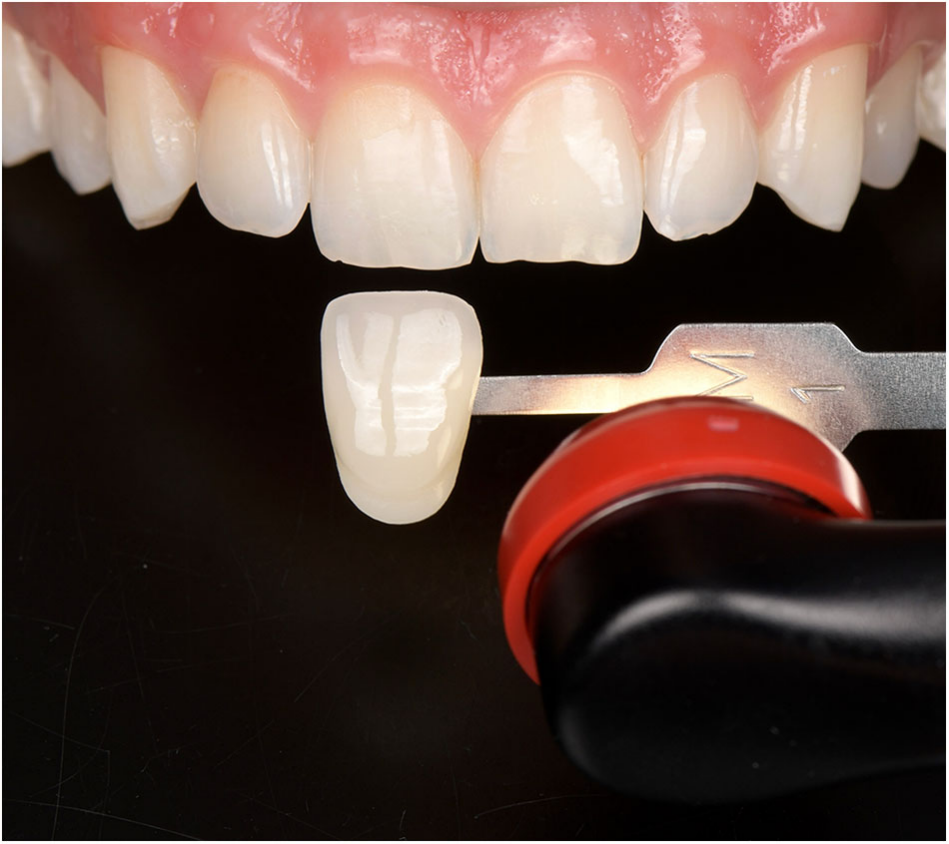
Use of a visible-light illumination lens. Example of a LCU used with a filtered visible-light illumination lens (Diffuser) to assist in clinical shade selection.

Finally, more anecdotal LCU experimental tips have been proposed to improve post-related cementation to deliver light deeper into root canals [163] and improve aligner attachment bonding [162]. Additionally, white light-filtered lens have been used to calibrate the light to 6500 K and enhance shade selection [181] (Fig. 12).

### Limitations of the search strategy

Despite the use of a structured search strategy and the SANRA recommendations, several limitations should be acknowledged. First, the three complementary literature searches conducted to perform this narrative review were limited to the single database (PubMed MEDLINE), English language publications, and a restricted time window starting in January 2000. This temporal restriction was chosen to avoid comparisons with older and currently less clinically relevant commercial LCUs while remaining sufficient to evaluate the evolution of these technologies over time. As a result, relevant evidence indexed exclusively in other databases, published in other languages, or available as gray literature may have been missed. In addition, the terminology related to light curing technologies and adjunctive optical features is not standardized and sometimes depends on the specific device or brand. Therefore, some studies may have been missed despite the iterative refinement of the keywords and the use of three complementary PubMed searches.

Second, the articles were selected by a single author. Although eligibility criteria were defined a priori and the author has a relevant background in adhesive dentistry and methodology, this approach may introduce selection and interpretation bias. Moreover, primarily for the sections on “light-cured resin composites for adhesive luting” and “optical adjunctive features”, which represent original analyses based on the knowledge summarized in this narrative review and for which highly specific search equations were deliberately not used during the design of the identification strategy, a significant number of articles were integrated on the basis of backward and forward citation tracking snowballing approaches and expert knowledge. While this approach may enhance conceptual coherence, it can reduce reproducibility compared with a fully systematic review process, which was not considered pertinent to address the objectives of the present work.

Finally, no formal risk of bias tool was applied, and the included evidence was methodologically heterogeneous, with a predominance of radiometric evaluations and in vitro studies, and limited long-term clinical outcome data. This limitation is particularly relevant for multifunctional LCUs that integrate fluorescence, transillumination, or beam-shaping accessories, for which independent comparative evidence remains scarce. Consequently, the conclusions of this review should be interpreted as practice-oriented guidance on the basis of the best available evidence rather than as definitive effect estimates.

## Conclusion

Light-curing should be approached as a controlled energy-delivery step rather than a simple, timed exposure. The transition to LED technology has improved ergonomics and versatility, but the clinical reliability of a light-curing unit cannot be inferred from the type of technology or the manufacturer-reported irradiance alone. Spectral compatibility with the photoinitiator system, the delivered radiant exposure, the spatial energy distribution, and appropriate clinical use by a trained practitioner are key determinants of curing reliability.

Within these limits, third-generation polywave LED LCUs remain conservative reference options in daily practice, particularly when the photoinitiator system of the resin-based material is unknown or when materials incorporating Norrish type I photoinitiators are used. In contrast, for camphorquinone-dominant materials, well-maintained second-generation monowave LED LCUs may provide comparable polymerization outcomes, provided that sufficient radiant exposure and adequate clinical conditions are ensured. In all cases, curing protocols should be adapted according to the clinical situation and safety considerations. Finally, multifunctional LCUs integrating fluorescence-aided identification and transillumination functions and beam-shaping accessories are appealing and may support minimally invasive workflows, but independent evidence remains limited.

## Data Availability

No new datasets were generated or analyzed during the preparation of this narrative review. Additional information related to the literature search and study selection process is available from the corresponding author upon reasonable request.
